# Local Administration of Ginkgolide B Using a Hyaluronan-Based Hydrogel Improves Wound Healing in Diabetic Mice

**DOI:** 10.3389/fbioe.2022.898231

**Published:** 2022-05-25

**Authors:** Limei Wang, Kedi Xia, Lu Han, Min Zhang, Jihuan Fan, Liu Song, Anqi Liao, Wenyu Wang, Jianfeng Guo

**Affiliations:** ^1^ Department of Pharmacy, Jilin Province FAW General Hospital, Changchun, China; ^2^ Department of Medical Administration, Jilin Province FAW General Hospital, Changchun, China; ^3^ Department of Ophthalmology and Otorhinolaryngology, Jilin Province FAW General Hospital, Changchun, China; ^4^ Department of Education and Science Services, Jilin Province FAW General Hospital, Changchun, China; ^5^ School of Pharmaceutical Sciences, Jilin University, Changchun, China; ^6^ Department of Thoracic Surgery, Jilin Province FAW General Hospital, Changchun, China

**Keywords:** hyaluronic acid, drug delivery, biomaterials, natural products, regenerative medicine

## Abstract

The delayed and incomplete healing of diabetic wounds remains a major concern of global healthcare. The complex biological processes within the diabetic wound, such as chronic inflammation, impaired blood vessel growth and immature collagen remodeling, dramatically cause the failure of current treatments. Thus, emerging therapeutic strategies are highly desirable. Ginkgolide B (GB, a natural product extracted from the leaves of Ginkgo biloba L.) has been applied in the treatment of cerebrovascular and cardiovascular disorders, which is mainly due to the anti-oxidative, anti-inflammatory and proliferative effects. In this study, the role of GB in facilitating the anti-inflammatory and pro-healing effects on diabetic wounds was for the first time confirmed using *in vitro*, *ex vivo* and *in vivo* experimental methods. As a consequence, GB was able to significantly achieve the activities of anti-inflammation, re-epithelialization, and pro-angiogenesis. Previously, a hydrogel has been developed using the high molecular weight hyaluronan (hyaluronic acid, HA) in our laboratory. In this study, this hydrogel was utilized *in vivo* for local administration of GB to the full-thickness wounds of diabetic mice. The resultant hydrogel formulation (HA-GB) resulted in the reduction of inflammation, the enhancement of re-epithelialization and angiogenesis, and the modulation of collagens from type III to type I, significantly promoting the healing outcome as compared with a commercially available wound dressing product (INTRASITE Gel). This study confirms a great therapeutic promise of HA-GB for the chronic wounds of diabetic patients.

## GRAPHICAL ABSTRACT

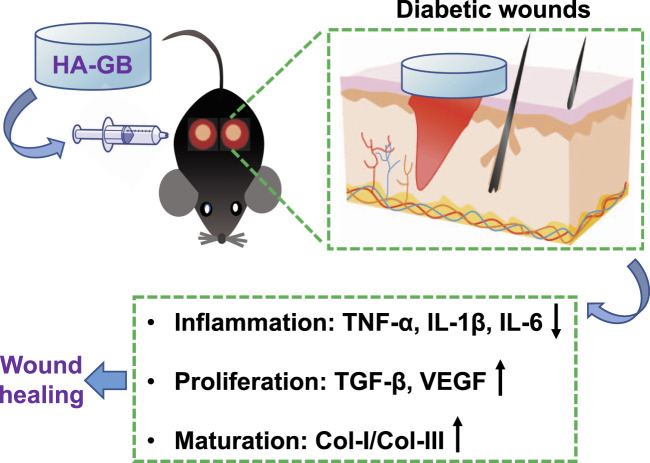

## Introduction

The healing process (i.e., hemostasis, inflammation, proliferation and maturation) generally proceeds efficiently within the injured site (Gurtner et al., 2008). However, certain pathological conditions (e.g., diabetes) may cause the delayed and incomplete wound healing (Eming et al., 2014). The chronic (non-healing) wound is one common complication of diabetes, and causes life-threatening infections for patients (Baltzis et al., 2014). Current treatments often fail, mainly due to the complex biological processes within the wound area (Greenhalgh, 2003), such as chronic inflammation, impaired angiogenesis (new blood vessel formation), and incomplete collagen remodeling. Recent strategies based on growth factors, cytokines and stem cells are designed and applied for diabetic wound healing (Zarei et al., 2018). However, these strategies usually target one single stage of healing process (Barrientos et al., 2014) (Teng et al., 2014) and lack appropriate delivery systems (Saghazadeh et al., 2018) (Oliva and Almquist, 2020). Therefore, the therapeutic efficacy is far from ideal, and emerging treatment alternatives are urgently required.

**FIGURE 1:**
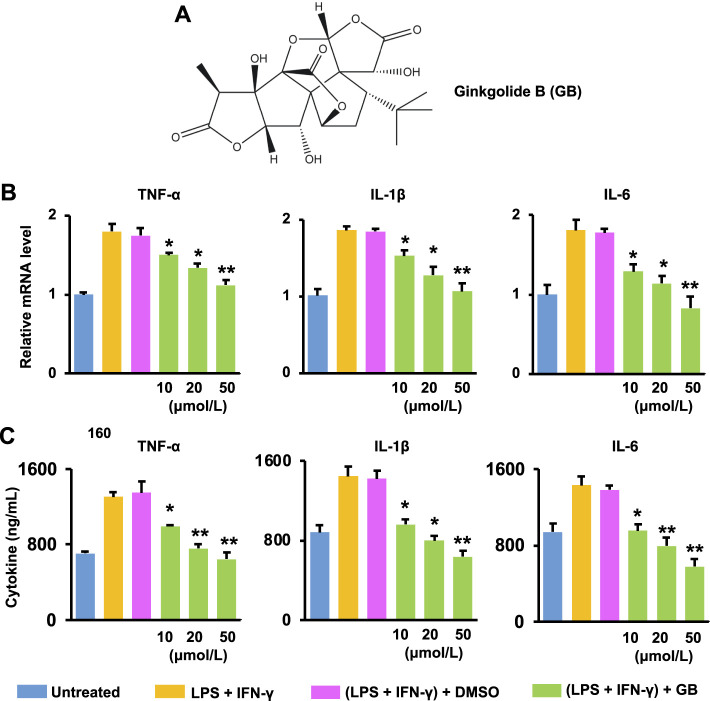
Ginkgolide B (GB) improves *ex vivo* anti-inflammatory effects. **(A)** GB’s chemical structure. **(B)** The mRNA level of three pro-inflammatory cytokines (TNF-α, IL-1β and IL-6) in LPS and IFN-γ co-stimulated BMDM treated with GB (10, 20, and 50 μmol/L) (*n* = 3; *p < 0.05 and **p < 0.01 vs. DMSO). **(C)** The release of TNF-α, IL-1β and IL-6 from co-stimulated BMDM with the treatment of GB (10, 20, and 50 μmol/L) (*n* = 3; *p < 0.05 and **p < 0.01 vs. DMSO).

Ginkgolide B (GB) is a terpenoid compound of the *Ginkgo biloba* L. leaf extracts (Figure 1A). It is known that GB acts as a potent competitive antagonist of the platelet activating factor (PAF) receptor, and has demonstrated neuroprotective and cardioprotective activities (Lv et al., 2011) (Xie et al., 2020) (Liu et al., 2020). These are mainly attributed to the anti-oxidant, anti-inflammatory and proliferative effects of GB (Gao et al., 2016) (Zheng et al., 2018) (Ge et al., 2020). Therefore, it is hypothesized that GB will achieve the repair and regeneration of diabetic wounds *via* targeting different stages of healing process. Here, the role of GB in facilitating the healing process was investigated using the *in vitro*, *ex vivo* and *in vivo* experiments. As a consequence, GB significantly resulted in the anti-inflammatory effect in mouse bone marrow-derived macrophages (BMDM), the proliferative activity in mouse fibroblasts (L929), and the pro-angiogenic efficacy in human umbilical vein endothelial cells (HUVEC). Previously, a hydrogel was produced in our laboratories by crosslinking a high molecular weight hyaluronan (also known as hyaluronic acid) (HA; *Mw* = 1,800 to 2,200 kDa) *via* adipic dihydrazide (Yang et al., 2021a). In this study, this hydrogel was utilized *in vivo* for local administration of GB to the full-thickness wounds of diabetic mice. The resulting hydrogel formulation (HA-GB) was able to profoundly speed up the wound closure, which mainly resulted from the alleviation of inflammatory mediators, the enhancement of re-epithelizing and pro-angiogenic factors, and the modulation of collagens from type III to type I. This study confirms for the first time that GB can promote the wound healing in diabetic mice by targeting the inflammation, proliferation and maturation phases. In addition, HA-GB profoundly enhanced the healing outcome when compared with a commercially available wound dressing product (namely INTRASITE Gel), indicating a great therapeutic potential of our strategy for diabetic wound healing.

## Materials and Methods

### Materials

Ginkgolide B (GB; CAS No. 15291-77-7; the chemical structure as shown in Figure 1A) was obtained from Shanghai TAUTO Company. The rest of reagents and materials were obtained in Sigma-Aldrich unless mentioned otherwise.

### Animals

Male C57BL/6 mice (∼5 weeks) were obtained in the Beijing Vital River Laboratory Animal Technology Company. Animals were allowed to acclimate for at least 7 days in the housing facility before the experiments, and every five mice were housed during the experiments. The experimental procedures were approved by Animal Ethics Committee of Jilin University.

### Cell Culture

Following the protocol as reported in (Shields et al., 2020), the BM cells were collected from the femurs with cold phosphate-buffered saline (PBS; Corning) and passed through the cell-strainer (70 μm; BD Falcon), prior to the centrifugation at 1,150 rpm for 5 min at 4°C. Following this, the lysis buffer (Yeasen Biotech, China) was used to remove erythrocytes, which was followed by the centrifugation at 4°C for 5 min at 1,150 rpm. Subsequently, 2 × 10^5^ of cells were cultured each well inside the 24-well plates using DMEM plus 10% fetal bovine serum (FBS), 1% penicillin-streptomycin (P/S) and 10 ng/ml macrophage colony-stimulating factor for 70–72 h. Then, cells were added with fresh medium as mentioned above for 48 h. Subsequently, cells were added with fresh DMEM plus 10% FBS, 1% P/S, lipopolysaccharide (LPS, 100 ng/ml) and interferon-γ (IFN-γ, 20 ng/ml) for 20–24 h, generating BMDM before the experiments as described below.

In addition, L929 and HUVEC were cultured using DMEM plus 10% FBS and 1% P/S and using RPMI-1640 plus 10% FBS and 1% P/S, respectively. In this study, all cells were grown at 37°C with 5% CO_2_ and 95% relative humidity.

### *Ex vivo* Anti-Inflammation

BMDM were cultured at a density of 2 × 10^5^ cells/well in 24-well plates for 1 day. Subsequently, GB ([c] = 10, 20, and 50 μmol/L) was added into BMDM for 20–24 h before the following experiments: 1) RT-qPCR. First-strand cDNAs were generated using TransScript^®^ First-Strand cDNA Synthesis SuperMix kit (TransGen Biotech, China). RT-qPCR was performed with TransStart^®^ Top Green qPCR SuperMix kit (TransGen Biotech) using StepOnePlus™ Real-Time PCR System (Thermo Scientific) under the conditions as previously reported (Guo et al., 2012). The information of tumor necrosis factor-α (TNF-α), interleukin 1β (IL-1β) and IL-6 primers was shown in Supplementary Table S1. 2) ELISA. The release of TNF-α, IL-1β and IL-6 in the culture supernatant was detected using the ELISA kits (LanpaiBIO, China) as previously reported (Guo et al., 2021) (Yu et al., 2020).

### *In vitro* Proliferation

L929 and HUVEC were cultured within 96-well plates (3,000 cells per well), respectively. Following 20–24 h, GB ([c] = 10, 20, 50, 100, and 300 μmol/L) was added into cells. After 1 day, cells were added by 200 μL of fresh medium and 20 μL of MTT solution (the stock concentration = 5 mg/ml in PBS) at 37°C for 4 h. The purple product was dissolved using DMSO prior to the measurement at 570 nm.

L929 and HUVEC were cultured in 6-well plates (4 × 10^5^ cells per well), respectively. Following 20–24 h, cells were incubated with GB ([c] = 10, 20, and 50 μmol/L) for 1 day before RT-qPCR (performed as above mentioned), in order to measure the expression of transforming growth factor beta (TGF-β) and vascular endothelial growth factor (VEGF). In addition, the release of TGF-β and VEGF in the culture supernatant was detected using the ELISA kits (LanpaiBIO, China).

The scratch experiment was performed as previously described (Sun, 2021). Briefly, L929 and HUVEC at a density of 4 × 10^5^ cells/well were cultured within 6-well plates, respectively. When ∼95–100% of confluence were reached, cells was washed with PBS and made for the “scratch.” Cells were incubated within serum free growth medium containing 40 μmol/L of GB. Following 1 day, the empty places before and after the incubation of GB were examined under the microscope (Olympus BX53).

### *In vivo* Healing Effects

A chemically induced diabetic mouse model was experimentally established as previously described, which may mimic type 1 diabetes and has been used for diabetic wound healing experiments (Yang et al., 2021a) (Chen et al., 2018) (Gan et al., 2019) (An et al., 2020). Briefly, animals were intraperitoneally injected with 55 mg/kg of streptozocin (STZ; 20% W/V) for 5 days, and the concentration of blood glucose in mice was measured using the blood glucose meter. Mice with >14 mmol/L blood glucose were considered diabetic (Gan et al., 2019) (Izumi et al., 2015). Subsequently, two full-thickness wounds (∼0.3 cm^2^) were made for each mouse using the disinfected surgical scissor into the dermis without disturbing the subdermal vasculature on the dorsal surface (Day 0). Mice were randomly grouped for the following experiments, and the blood glucose concentration was measured during the experiments to confirm >14 mmol/L in mice (Supplementary Figure S1).

The hydrogel was prepared by crosslinking sodium hyaluronate (*Mw* = 1,800 to 2,200 kDa) *via* adipic dihydrazide as previously described in our laboratory (Yang et al., 2021a). To form the GB-loaded formulation (HA-GB), 1 ml of GB solution ([c] = 250, 500, 1,000, and 2,000 μmol/L in PBS with 1% propylene glycol) was loaded with 0.08 g of freeze-dried hydrogel (this achieved 8% hydrogel, and GB was fully loaded at this concentration). In addition, at Day 0, 2, and 4, the wounds were topically treated with/without 100 μl of blank hydrogel, 100 μl of INTRASITE Gel or 100 μl of HA-GB, and subsequently were covered by the 3M dressing. The dressing was removed at Day 6. The healing rate = (1-Sn/S0) × 100%, in which Sn is the surface area on chosen days, S0 is the surface area on Day 0. The wounds were dissected (no healthy tissue was part of the resected area) for:

1) H&E staining assay. The samples were fixed by 4% paraformaldehyde, embedded using the paraffin, and sectioned for the hematoxylin-eosin (H&E) stain experiment (Qin et al., 2018). The infiltration of inflammatory cells, induction of epidermal hyperplasia and formation of blood vessels were evaluated within the H&E-stained sections (5 μm) and quantified as described previously (Yang et al., 2021a) (Gao et al., 2019). Briefly, the number (N) of positive cells and the area 1) were detected using Image-Pro Plus 6.0 (Media Cybernetics). The inflammatory cell infiltration of treated groups was determined as N/a corrected to the untreated group. Moreover, the mean of epidermal thickness was calculated based on three areas in the epidermis layer using Image-Pro Plus 6.0. The epidermal hyperplasia of treated groups was determined as the mean of epidermal thickness corrected to the untreated group. Furthermore, the blood vessel formation was determined based on the mean of new blood vessels. These were performed by two professional pathologists separately. 2) Masson’s trichrome staining assay. The sections (5 μm) were used for the Masson’s trichrome stain experiment (Gao et al., 2019). The collagen deposition was determined within the Masson’s trichrome-stained sections as previously described (Yang et al., 2021a) (Gao et al., 2019). 3) RT-PCR and ELISA assays. The samples were homogenized and centrifuged at 12,000 rpm for 25–30 min at 4°C. The RT-qPCR and ELISA experiments for inflammatory and growth factors were performed as described above. 4) Western blotting assay. The concentration of proteins (collected above) was measured by the BCA kit (TransGen Biotech), and a sample containing ∼40 μg of proteins was used in SDS-PAGE (90 V for 2 h). The proteins were subsequently transferred to the PVDF membrane (Millipore) at 180 mA for 2 h. The membrane was added with primary antibodies at 4°C overnight, and was subsequently added with secondary antibodies at room temperature for 1 h (see the information of antibodies in Supplementary Table S2). The enhanced chemiluminescence solution (GE Healthcare) was applied to identify the proteins.

### Statistical Analysis

Data was shown as a mean ± standard deviation. The unpaired Student’s t-test was applied to analyze the significance of two groups. The significance of three or more groups was analyzed using the one-way ANOVA under the Bonferroni’s Post-Hoc test. In this study, *p* < 0.05 was considered statistically significant.

## Results and Discussion

### Ginkgolide B Improves Anti-Inflammatory Effects

The healing process proceeds orderly and efficiently inside the wound site at the onset of injuries (Gurtner et al., 2008). However, the healing is often “stalled” in the inflammation phase, which is mainly caused by an elevated production of pro-inflammatory mediators within the diabetic wounds (Salazar et al., 2016). GB (Figure 1A), a natural product extracted from the leaves of *Ginkgo biloba L.*, exerts the anti-inflammatory activity in protecting against inflammation-associated disorders (e.g., stroke and cardiomyopathy) (Shu et al., 2016) (Li et al., 2021a). In this study, the anti-inflammatory effects of GB were assessed *ex vivo* using LPS and IFN-γ co-stimulated BMDM (Figures 1B,C). Results of Figure 1B show that the mRNA level of TNF-α, IL-1β and IL-6 (three pro-inflammatory cytokines critically responsible for the inflammation phase) in co-stimulated BMDM was significantly (*p* < 0.05 and 0.01) alleviated by GB in a concentration-dependent manner. When the concentration of GB was reached to 50 μmol/L, the mRNA level of these cytokines in the co-stimulated BMDM was similar to that recorded in the non-stimulated BM cells (Figure 1B). Accordingly, the release of these cytokines was also significantly (*p* < 0.05 and 0.01) decreased by GB (Figure 1C). The co-stimulated BMDM can highly mimic M1 macrophages, which are a prominent pro-inflammatory cell type during the inflammation phase in diabetic wound (Kim and Nair, 2019). Therefore, the results of Figures 1B,C demonstrate that GB can potentially ameliorate the chronic inflammation for protecting against diabetic wounds.

### Ginkgolide B Promotes Proliferative Activities

When the healing process moves forwards, the proliferation phase proceeds with the closure of injured surface (re-epithelialization), the formation of blood vasculature (angiogenesis), and the development of granulation tissue (Martin and Nunan, 2015). However, diabetic wounds are usually evident with the failure in these steps (Guo and Dipietro, 2010).

**FIGURE 2:**
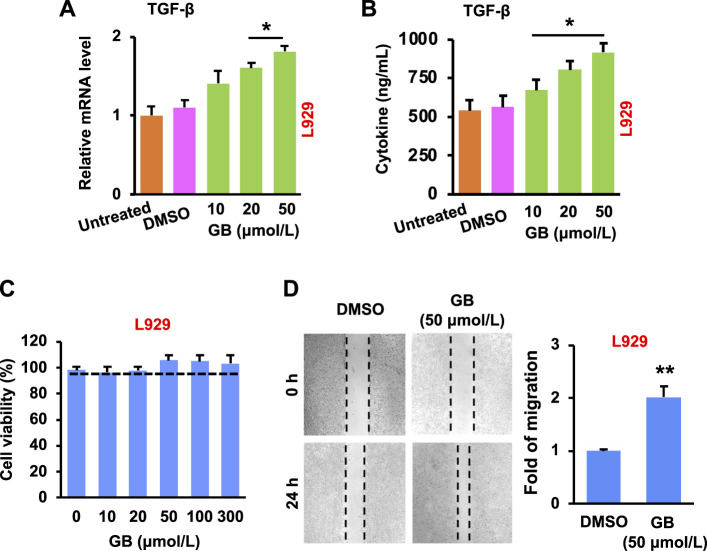
GB promotes *in vitro* proliferative activity of fibroblasts. **(A)** The level of TGF-β mRNA in L929 following GB treatment (10, 20, and 50 μmol/L) (*n* = 3; *p < 0.05). **(B)** The release of TGF-β protein in the culture supernatant of L929 following GB treatment (10, 20, and 50 μmol/L) (*n* = 3; *p < 0.05). **(C)** Cytotoxicity of L929 after the treatment of GB (10, 20, 50, 100, and 300 μmol/L; 0 = DMSO alone). **(D)** The empty places before and after treatment of GB (50 μmol/L) were measured for the fold of migration (the magnification = ×10) (*n* = 3; **p < 0.01).

The re-epithelialization is highly relied on the production of extracellular matrix (ECM) components (e.g., collagens, glycosaminoglycans and proteoglycans) (Xue and Jackson, 2015). The ECM components are mainly produced by fibroblasts (one of the most abundant cells in the wound area (Bainbridge, 2013)), and the fibroblasts can be activated by transforming growth factor beta (TGF-β) (Barrientos et al., 2008). Results in Figures 2A,B show that GB significantly (*p* < 0.05) elevated the expression of TGF-β mRNA and protein in L929 (a mouse fibroblastic cell line) in a concentration-dependent manner. In addition, no significant cytotoxicity in L929 was caused by GB under the concentrations tested (10–300 μmol/L), while the cell growth was slightly enhanced by GB at > 20 μmol/L (Figure 2C). Moreover, GB was able to significantly (*p* < 0.01) facilitate the migration of L929 (Figure 2D). Therefore, these results suggest that GB is able to improve the activity of TGF-β in fibroblasts for accelerating the wound closure.

**FIGURE 3:**
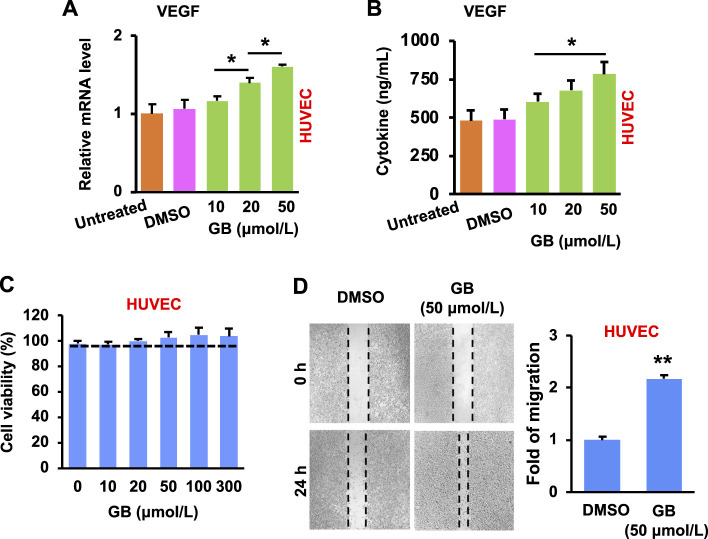
GB promotes *in vitro* proliferative activity of endothelial cells. **(A)** The level of VEGF mRNA in HUVEC following GB treatment (10, 20, and 50 μmol/L) (*n* = 3; *p < 0.05). **(B)** The release of VEGF protein in the culture supernatant of HUVEC following GB treatment (10, 20, and 50 μmol/L) (*n* = 3; *p < 0.05). **(C)** Cytotoxicity of HUVEC after the treatment of GB (10, 20, 50, 100, and 300 μmol/L). **(D)** The empty places before and after treatment of GB (50 μmol/L) were measured for the fold of migration (*n* = 3; **p < 0.01).

The development of new blood vessels is closely regulated by growth factors (e.g., vascular endothelial growth factor, VEGF) that stimulate endothelial cells of the existing vessels (Barrientos et al., 2008). The activated endothelial cells migrate towards and grow inside the injured area, resulting in the formation of vascular network (Guo and Dipietro, 2010). Results of Figures 3A,B show that GB significantly (*p* < 0.05) increased the expression of VEGF mRNA and protein in HUVEC (a human umbilical vein endothelial cell line) in a concentration-dependent manner. In addition, GB caused no significant cytotoxicity of HUVEC under the concentrations tested (10–300 μmol/L), but slightly enhanced the cell growth when the concentration was >20 μmol/L (Figure 3C). Moreover, GB was able to significantly (*p* < 0.01) facilitate the migration of HUVEC (Figure 3D). These results imply that GB can promote the activity of VEGF in endothelial cells for exerting the new blood vessel formation.

The formation of granulation tissue (a type of connective tissue) is closely concomitant with the development of ECM and new blood vessels (Sorg et al., 2017). Results of Figures 2, 3 confirm the capacity of GB for the development of ECM and angiogenesis by the production of growth factors and by the stimulation of fibroblasts and endothelial cells. These demonstrate the potential of GB for the formation of granulation tissue. Taken together, GB can potentially fulfill the proliferation phase of diabetic wounds.

### Ginkgolide B Achieves Diabetic Wound Healing

**FIGURE 4:**
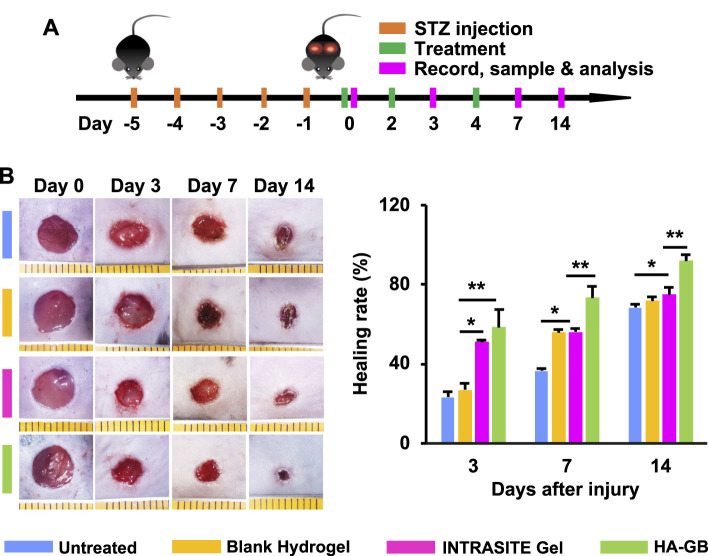
HA-GB facilitates *in vivo* diabetic wound healing. **(A)** Treatment, sample and analysis schematic. **(B)** The wound images following treatment of HA-GB (1,000 μmol/L). Healing rate (%) (*n* = 8; *p < 0.05 and **p < 0.01).

The capacity of GB for improving diabetic wound healing was further confirmed in diabetic mice with full-thickness wounds. The diabetes in mice is chemically induced due to the ablation of β-cells by STZ (a nitrosourea analogue, one of the most potent diabetogenic chemicals) (Al-Awar et al., 2016), and such a diabetic mouse model has been substantially used in the field of diabetes research (Pandey and Dvorakova, 2020). Recently, a variety of drug delivery systems have been designed and applied for delivery of therapeutic agents in wound repair and regeneration (Manconi et al., 2018) (Orsu et al., 2021) (Li et al., 2021b) (Rathinavel et al., 2021). Previously, a hydrogel was produced in our laboratory by crosslinking a HA polymer (*Mw* = 1,800–2,200 kDa) (Yang et al., 2021a). This hydrogel possesses favorable physicochemical characteristics in terms of porous 3D structure, enzymatic hydrolysis resistance, and sustained release (Yang et al., 2021a). In this study, this hydrogel was used *in vivo* for topical application of GB to the full-thickness wounds of diabetic mice (Figure 4, Supplementary Figure S1). The resultant formulation (HA-GB) demonstrated the full loading and sustained release of GB (Supplementary Figure S2). The healing rate of HA-GB (containing 250, 500, 1,000 and 2,000 μmol/L of GB) was assessed to investigate the *in vivo* dose of GB (Supplementary Figure S3). Results of Supplementary Figure S3 indicate that HA-GB at 1,000 μmol/L of GB significantly accelerated the wound healing (*p* < 0.05); therefore, 1,000 μmol/L of GB were chosen for the *in vivo* experiments.

**FIGURE 5:**
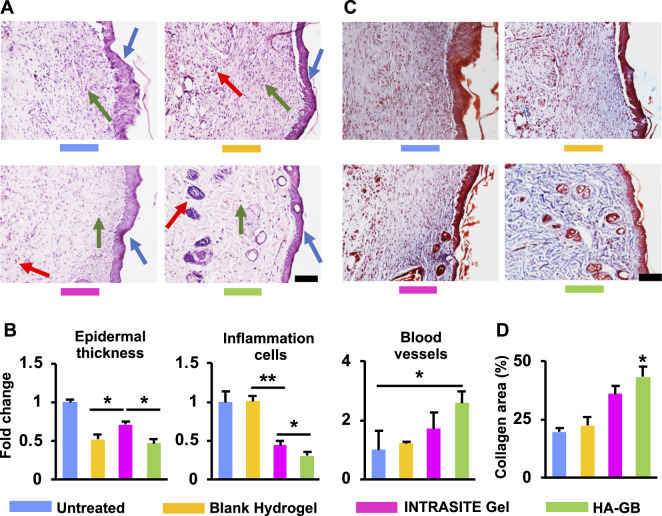
The histological analysis. **(A)** In the H&E images on Day 7 (scale = 20 μm), the inflammatory cells, epidermal hyperplasia and new blood vessels were indicated by the green, blue and red arrows, respectively. **(B)** The quantification of Figure 5A (*n* = 6; *p < 0.05 and **p < 0.01). **(C)** The Masson’s trichrome images on Day 14 (scale = 20 μm) (red area = keratin, muscle fibers and cytoplasm; blue area = collagens). **(D)** The quantification of Figure 5C (*n* = 6; *p < 0.05).

The diabetic wounds were topically administrated with various treatments at Day 0, 2 and 4 (Figure 4A). As shown in Figure 4B, INTRASITE Gel remarkably speeded up the closure of injured site as compared with blank hydrogel (*p* < 0.05, Day 3), while HA-GB could further promote the healing outcome as compared with INTRASITE Gel (*p* < 0.01, Day 3, 7, and 14) (Figure 4B). As a result, HA-GB significantly (*p* < 0.05) achieved the reduction of inflammatory cell infiltration (indicated by green arrow), the amelioration of epidermal hyperplasia (indicated by blue arrow) and the enhancement of angiogenesis (indicated by red arrow) as compared with the other groups (Day 7) (Figures 5A,B). Furthermore, the deposition of collagens was also significantly (*p* < 0.05) achieved by HA-GB as compared with the other groups (Day 14) (Figures 5C,D). Therefore, results of Figures 4, 5 demonstrate that HA-GB was able to accelerate the diabetic wound healing, which was mainly due to the capacity of GB for suppressing the inflammatory cell infiltration, alleviating the epidermal hyperplasia, improving the angiogenesis, and facilitating collagen deposition.

**FIGURE 6:**
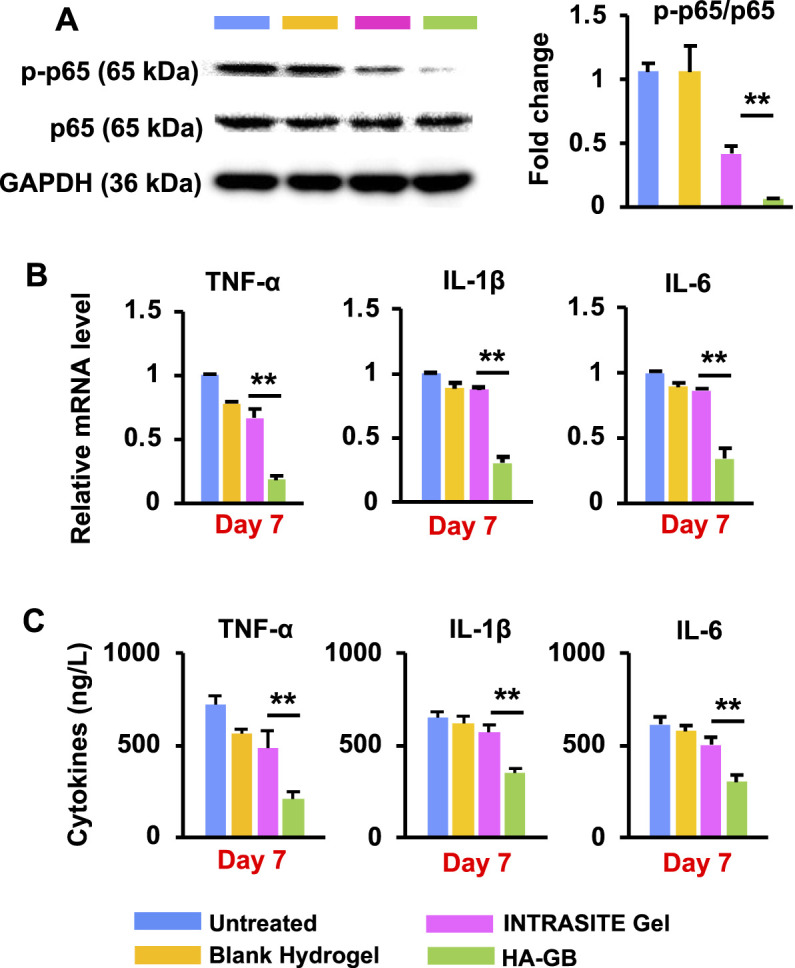
*In vivo* anti-inflammatory effects of HA-GB. **(A)** The phosphorylation of p65 in diabetic wounds and the quantification of protein expression following HA-GB treatment (1,000 μmol/L) (*n* = 4; **p < 0.01). **(B)** The mRNA level of three pro-inflammatory cytokines (TNF-α, IL-1β and IL-6) in diabetic wounds following HA-GB treatment (*n* = 4; **p < 0.01). **(C)** The release of three pro-inflammatory cytokines in diabetic wounds following HA-GB treatment (*n* = 4; **p < 0.01).

It has been reported that the nuclear factor kappa-B (NF-κB) pathway is continuously activated within non-healing wounds; consequently, the overexpression of inflammation cytokines (e.g., TNF-α, IL-1β and IL-6) causes the wounds trapped in the chronic inflammatory state (Zhao et al., 2016). In this study, the activity (phosphorylation) of p65 (a key component in the NF-κB transcription factor complex) was significantly suppressed by HA-GB in diabetic wounds relative to blank hydrogel and INTRASITE Gel (*p* < 0.05, Figure 6A). Accordingly, HA-GB was capable of significantly (*p* < 0.05) decreasing the level of TNF-α, IL-1β and IL-6 mRNA when compared to the controls (Figure 6B). HA-GB also profoundly inhibited the production of these pro-inflammatory cytokines (*p* < 0.05, Figure 6C). These results indicate the *in vivo* function of GB in the downregulation of pro-inflammatory NF-κB pathway for promoting the inflammation phase of diabetic wounds, which was also confirmed by the inhibition of the inflammatory cell infiltration (Figures 5A,B).

**FIGURE 7:**
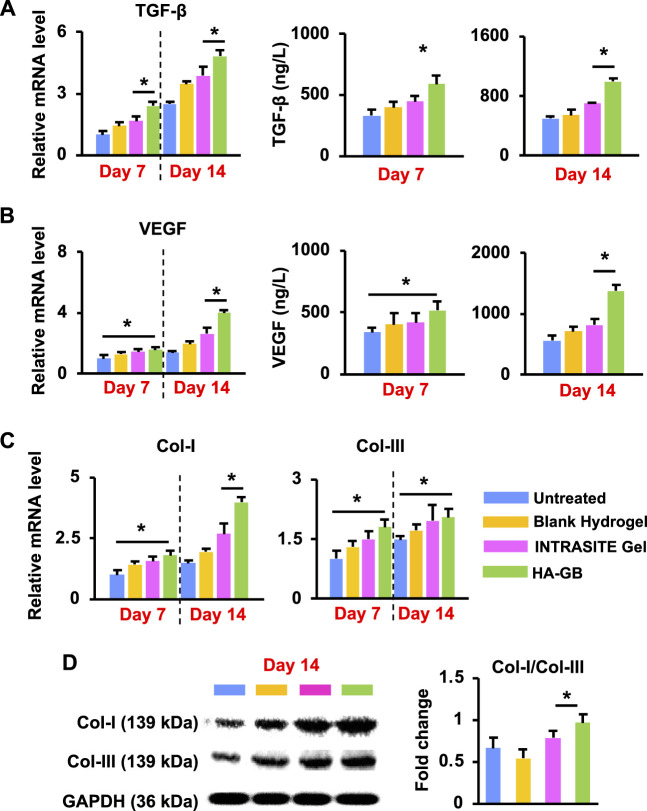
*In vivo* pro-healing effects of HA-GB. **(A)** The mRNA and protein levels of TGF-β in diabetic wounds following HA-GB treatment (1,000 μmol/L) at Day 7 and 14 (*n* = 4; *p < 0.05). **(B)** The mRNA and protein levels of VEGF in diabetic wounds following HA-GB treatment at Day 7 and 14 (*n* = 4; *p < 0.05). **(C)** The mRNA level of Col-I and Col-III in diabetic wounds following HA-GB treatment (*n* = 4; *p < 0.05). **(D)** The protein expression of Col-I and Col-III in diabetic wounds following HA-GB treatment (*n* = 3; *p < 0.05).

As mentioned above, the re-epithelization, angiogenesis and collagen remodeling in the proliferation and maturation stages are closely regulated by growth factors such as TGF-β and VEGF (Yang et al., 2021b). In this study, HA-GB was capable of significantly increasing the level of TGF-β mRNA within diabetic wounds on Day 7 and 14 (*p* < 0.05) (Figure 7A); accordingly, the release of TGF-β protein could also be dramatically upregulated by HA-GB (*p* < 0.05, Figure 7A). These resulted in the amelioration of epidermal hyperplasia (a key indicator for re-epithelization efficacy) (Figures 5A,B). Moreover, the mRNA and protein levels of VEGF were also significantly increased by HA-GB (Figure 7B), facilitating the development of new blood vessels (Figures 5A,B). It has been reported that the transition of collagens from type III (Col-III) to type I (Col-I) is a hallmark during the granulation tissue formation, and the failure of collagen remodeling may cause an excessive scar (Tracy et al., 2016). As shown in Figure 7C, after the HA-GB treatment, the expression of Col-I and Col-III was significantly improved (*p* < 0.05), which was also confirmed by the deposition of collagens as evident in Figures 5C,D. More importantly, the ratio of Col-I to Col-III was significantly increased with the treatment of HA-GB (*p* < 0.05) (Figure 7D), demonstrating the successful transition of collagens from type III to type I. Taken together, results of Figure 7 confirm that HA-GB can promote the wound healing in diabetic mice due to the capacity of GB for fully targeting the inflammation, proliferation and maturation phases.

## Conclusion

**FIGURE 8:**
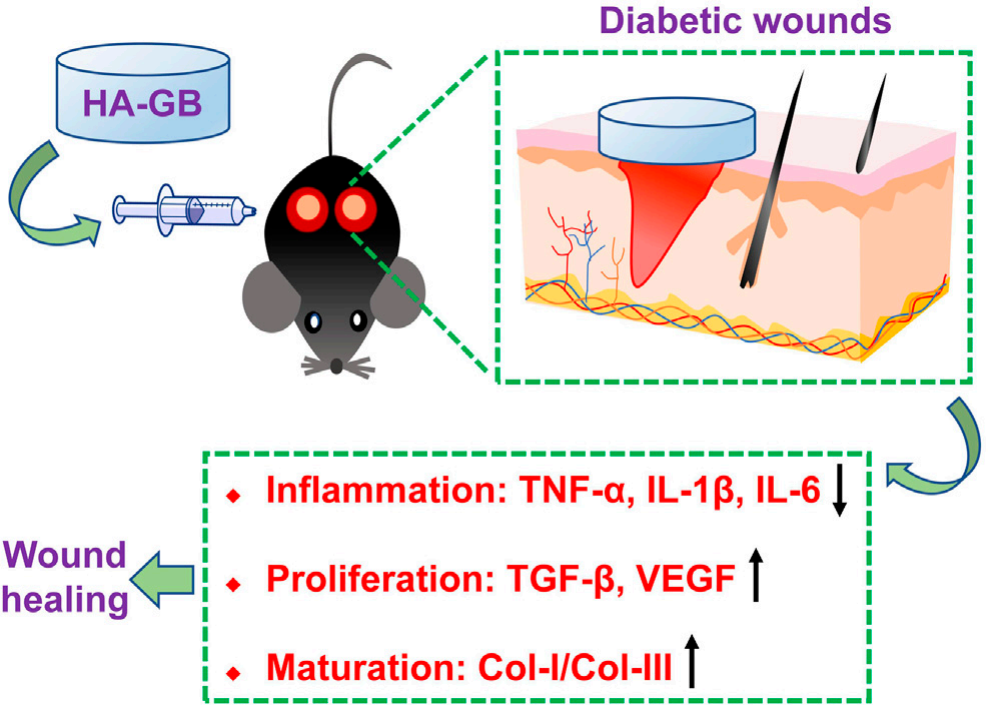
Local administration of Ginkgolide B using a hyaluronic acid-based hydrogel improves wound healing in diabetic mice.

Increasing understanding in cellular and molecular mechanisms for the chronic wounds has significantly revolutionized the development of wound healing strategies (Guo and Dipietro, 2010) (Sun et al., 2014). Emerging growth factor, cytokine and stem cell therapies have been designed for the application of chronic wound healing (Zarei et al., 2018). Despite these promises, therapeutic outcome is still low, mainly due to the fact that these therapies generally target one single phase of healing process (Barrientos et al., 2014) (Teng et al., 2014) and lack appropriate delivery systems (Saghazadeh et al., 2018) (Oliva and Almquist, 2020). In this study, Ginkgolide B (GB), a natural product extracted from the leaves of *Ginkgo biloba L.*, achieved *in vitro*, *ex vivo* and *in vivo* anti-inflammatory, re-epithelializing, pro-angiogenic, and collagen-remodeling effects (Figure 8), confirming the capacity of GB for satisfying the inflammatory, proliferative and maturation phases of healing process. Recent developments of drug delivery systems have significantly advanced the administration of therapeutic agents for chronic wounds (Voss et al., 2018; Hsu et al., 2019; Morey and Pandit, 2018; Chin et al., 2019). In this study, a crosslinked hyaluronan-based hydrogel was used *in vivo* for topical application of GB, and the resultant hydrogel formulation (HA-GB) profoundly speeded up the closure of diabetic full-thickness wounds (Figure 8). More importantly, HA-GB significantly improved the healing outcome relative to INTRASITE Gel, confirming the great promise of our strategy in the clinical application for diabetic wound repair and regeneration.

## Data Availability

The raw data supporting the conclusion of this article will be made available by the authors, without undue reservation.
