# Supplementary material for: Local Administration of Ginkgolide B Using a Hyaluronan-Based Hydrogel Improves Wound Healing in Diabetic Mice
Source: Front Bioeng Biotechnol. 2022 May 25;10:898231. doi: 10.3389/fbioe.2022.898231 (PMC9174682; doi:10.3389/fbioe.2022.898231)
Supplement: Supplementary file 1 [file DataSheet1.docx]

*Supporting Information for*

**Local administration of Ginkgolide B using a hyaluronan-based hydrogel improves wound healing in diabetic mice**

Limei Wang ^1 #^, Kedi Xia ^1 #^, Lu Han ^2^, Min Zhang ^3^, Jihuan Fan ^4^, Liu Song ^5^, Anqi Liao ^5^, Wenyu Wang ^6^ *, and Jianfeng Guo ^5^ *

^1^Department of Pharmacy, Jilin Province FAW General Hospital, Changchun 130011, China

^2^Department of Medical Administration, Jilin Province FAW General Hospital, Changchun 130011, China

^3^Department of Ophthalmology and Otorhinolaryngology, Jilin Province FAW General Hospital, Changchun 130011, China

^4^Department of Education and Science Services, Jilin Province FAW General Hospital, Changchun 130011, China

^5^School of Pharmaceutical Sciences, Jilin University, Changchun 130021, China

^6^Department of Thoracic Surgery, Jilin Province FAW General Hospital, Changchun 130011, China

^#^Limei Wang and Kedi Xia shared first authorship

*Corresponding authors: wangwenyufaw@163.com (W. Wang), jguo@jlu.edu.cn (J. Guo)

**Figure S1**. The concentration of blood glucose in STZ-treated mice used in the wound healing experiments (n = 8; used in Fig. 4). During the experitments, the blood glucose of STZ-treated mice used in the untreated group, blank HA, INTRASITE gel and HA-GB was identified over 17 mmol/L, which was significantly higher than that of control mice (without STZ treatment).

**Figure S2**. The release rate of GB from HA-GB (n = 3). In this experiment, 1 mL of GB solution (10 mg/mL; that was 23500 μmol/L) was added to 0.08 g of freeze-dried HA, achieving HA-GB (8% hydrogel, and the GB solution was fully absorbed by freeze-dried HA at this condition, theoretically, GB was fully loaded into the hydrogel). The HA-GB was incubated in 5 mL PBS (containing 1% propylene glycol) at 37 ^o^C with gentle shaking. The amount of GB released from HA-GB into the solution at different time points was measured using UV-vis spectrophotometer (220 nm) to determine the release rate. Notably, the release rate of GB from HA-GB was not affected by the concentration of GB solution, as similar release results were also achieved when the GB solution was 7.5 mg/mL or 15 mg/mL.

**Figure S3**. The healing rate (%) of diabetic mice treated with HA-GB at different doses of GB on Day 3, 7 and 14 (n = 4). * *p* < 0.05 and ** *p* < 0.01 relative to the untreated group. It is worth noting that HA-GB at concentrations of 50, 100 (data not shown) and 250 μmol/L could not significantly (*p* > 0.05) facilitated the wound healing as compared to untreated control. In contrast, HA-GB at concentrations of 500 and 1000 μmol/L significantly (*p* < 0.05 and < 0.01) improved the wound healing as compared to untreated control. The wound healing efficacy was less effective using HA-GB at concentrations of 2000 μmol/L as compared to HA-GB at concentrations of 500 and 1000 μmol/L (reason awaits future investigation). In addition, the animal body weight was not significantly affected following the treatment of HA-GB at different concentrations as compared to untreated group (data not shown), indicating no toxicity caused *in vivo*.

**Table S1**. Primers used for RT-PCR in the study.

| **Primer** | **Sequence (5’ to 3’)** |
| --- | --- |
| TNF-α-forward | CAGGCGGTGCCTATGTCTC |
| TNF-α-reverse | CGATCACCCCGAAGTTCAGTAG |
| IL-1β-forward | GAAATGCCACCTTTTGACAGTG |
| IL-1β-reverse | GAAATGCCACCTTTTGACAGTG |
| IL-6-forward | CCTCTCTGCAAGAGACTTCCATCCA |
| IL-6-reverse | AGCCTCCGACTTGTGAAGTGGT |
| VEGF-forward | GCACATAGAGAGAATGAGCTTCC |
| VEGF-reverse | CTCCGCTCTGAACAAGGCT |
| TGF-β-forward | CCACCTGCAAGACCATCGAC |
| TGF-β-reverse | CTGGCGAGCCTTAGTTTGGAC |
| COL-I-forward | AAGGAGTTTCATCTGGCCCT |
| COL-I-reverse | AGCAGGTCCTTGGAAACCTT |
| COL-III-forward | CTGTAACATGGAAACTGGGGAAA |
| COL-III-reverse | CCATAGCTGAACTGAAAACCACC |
| GAPDH-forward | GAGAGGCCCTATCCCAACTC |
| GAPDH-reverse | GATTGAGCCTGCTTCACCTC |

**Table S2**. Antibodies used for western blotting in the study.

| **Antibody** | **Company** | **Catalog No.** | **Dilution** |
| --- | --- | --- | --- |
| Anti-p65 | Immunoway | YM3111 | 1:2000 |
| Anti-p-p65 | Immunoway | YP0189 | 1:2000 |
| Anti-COL-I | Affinity | AF7001 | 1:2000 |
| Anti-COL-III | Affinity | AF0136 | 1:2000 |
| Anti-GAPDH | Affinity | AF7021 | 1:20000 |
| Goat Anti-Rabbit lgG (H+L) HRP | Immunoway | RS0002 | 1:20000 |
